# Dr. James W. Craig

**Published:** 1891-09

**Authors:** 


### Obituary.

---

DR. JAMES W. CRAIG, of Churchville, Monroe county, N. Y., died at his home on Friday, June 26, 1891, aged 65 years. Dr. Craig was one of the best known and ablest physicians in his region, and his friends, neighbors, patients and relatives will keenly feel and deeply lament his demise. His death resulted from septicemia, caused by the prick of a pin in one of his fingers, which occurred on the 17th of May last. Dr. Craig was born November 8, 1825, in the town of York, Livingston county. He was the son of William and Jane Craig, of Scotch descendants, received an academic education, and attended schools in Le Roy and Geneseo, under the tutorage of Rev. Donald D. McCall. He studied medicine with his uncle, J. R. Craig, M. D., of Mumford, and attended medical lectures at the University of Pennsylvania, at the University of Buffalo, and at the Jefferson Medical College, of Philadelphia. He commenced practice in Churchville, April 1, 1851, and had continued the same until a short time before his death.

He was married to Sarah S. Butterfield, of Caledonia, September 2, 1851. Six daughters were the result of this marriage, two dying in infancy. The four living are: Mrs. Jennie, wife of Rev. John B. Jones, of Tama, Iowa; Dr. Sarah Craig Buckley, now in Kyoto, Japan; Dr. Marian Craig, Lake avenue, Rochester, and Dr. Anna Craig, associated with Dr. Marian. His second marriage took place May 7, 1890, to Mrs. Almira Doty Borst, with whom he was living at the time of his death. Dr. Craig was honored by many responsible positions at the hands of his fellow-physicians, and had been a Curator of the Buffalo University Medical College for many years.

